# Changes in Surface Characteristics of BOPP Foil after Treatment by Ambient Air Plasma Generated by Coplanar and Volume Dielectric Barrier Discharge

**DOI:** 10.3390/polym13234173

**Published:** 2021-11-29

**Authors:** Petra Šrámková, Zlata Kelar Tučeková, Michal Fleischer, Jakub Kelar, Dušan Kováčik

**Affiliations:** 1 Department of Physical Electronics, CEPLANT—R&D Centre for Plasma and Nanotechnology Surface Modifications, Faculty of Science, Masaryk University, Kotlářská 2, 611 37 Brno, Czech Republic; petra.sramkova@mail.muni.cz (P.Š.); zlata.tucekova@mail.muni.cz (Z.K.T.); michal.fleischer@mail.muni.cz (M.F.); jakub.kelar@mail.muni.cz (J.K.); 2 Department of Experimental Physics, Faculty of Mathematics, Physics and Informatics, Comenius University in Bratislava, Mlynská dolina, 842 48 Bratislava, Slovakia

**Keywords:** BOPP foil, DCSBD, VDBD, surface wettability, adhesion, ageing, surface functionalization

## Abstract

Biaxially oriented polypropylene (BOPP) is a highly transparent polymer defined by excellent mechanical and barrier properties applicable in the food packaging industry. However, its low surface free energy restricts its use in many industrial processes and needs to be improved. The presented study modifies a BOPP surface using two different atmospheric-pressure plasma sources operating in ambient air and capable of inline processing. The volume dielectric barrier discharge (VDBD) and diffuse coplanar surface barrier discharge (DCSBD) were applied to improve the wettability and adhesion of the 1–10 s treated surface. The changes in morphology and surface chemistry were analyzed by SEM, AFM, WCA/SFE, and XPS, and adhesion was evaluated by a peel force test. Comparing both plasma sources revealed their similar effect on surface wettability and incorporation of polar functional groups. Additionally, higher surface roughness in the case of VDBD treatment contributed to slightly more efficient adhesion in comparison to DCSBD. Although we achieved comparable results for both plasma sources in the term of enhanced surface wettability, degree of oxidation, and stability of induced changes, DCSBD had less effect on the surface deterioration than VDBD, where surface structuring caused an undesirable haze.

## 1. Introduction

Biaxially oriented polypropylene (BOPP) is produced from melted polypropylene stretched in both transverse directions, producing molecular chains oriented in cross directions. Stretching into both directions ensures the significant improvement of its properties including enhanced barrier properties, increased toughness, and stiffness [1]. These properties, along with high transparency, make BOPP an excellent and cost-effective material in food [2,3] and tobacco packaging, but also in high energy density applications, e.g., capacitor production [4]. The BOPP’s low surface free energy, however, hinders processes where good printability, adhesion, or improved wettability are required. The treatment by cold atmospheric-pressure plasma provides a popular solution for the surface activation of polymers and other thermally sensitive materials. However, the overturning and migration of these surface functional groups into a volume of material result in so-called hydrophobic recovery. This phenomenon often appears within days, and a loss of improved properties can be observed within a month on most BOPP substrates [5,6]. Thus, stable surface functionalization and adhesion improvement are required for industrially produced BOPP, often stored before further processing.

Dielectric barrier discharges (DBD) of various geometries are often used as the atmospheric-pressure nonequilibrium plasma sources for inline industrial applications. Volume DBD (VDBD), so-called “industrial corona”, and diffuse coplanar surface barrier discharge (DCSBD) with a concavely curved electrode system suitable for roll-to-roll arrangement are often used for flexible large-area material treatment such as foils [7], paper, [8] and nonwoven textiles [9]. The treatment by atmospheric-pressure air DBD introduces polar functional groups providing hydrophilicity and an increase of surface free energy of the polymer surface. Industrial corona represents the most widely used plasma source in commercial use due to its simple operation in ambient air at the atmospheric pressure, fast speed, as well as short treatment times sufficient for material activation. However, corona discharge comprises hot filamentary microdischarges, which often cause thermally sensitive polymers’ deterioration due to the perpendicular orientation of randomly distributed microdischarges towards the treated surface. In contrast to industrial corona, DCSBD enables generation of plasma consisting of microdischarges, where diffuse parts are intensified, while the filamentary elements are suppressed and parallel to the treated surface. The resulting diffuse plasma is thin, homogeneous, more effective, and less harmful to sensitive polymer materials.

Until now, the efficiency of surface activation of DCSBD plasma has been investigated on several polymer substrates. The high power density of such plasma ensured the improved wettability in the case of polyesters such as PEN [10], PET [11], PLA [12], or polycarbonate [13], as well as PMMA [14], even after 1 s of treatment. Moreover, our recent study considering the surface changes of PA 6 after DCSBD plasma treatment demonstrated the high potential of this technology to be part of industrial systems due to its very fast surface activation (0.25 s) and long-term preservation of the achieved properties [7]. The largest group of produced polymers worldwide represented by polyolefins were subjected to DCSBD plasma treatment in several studies [15,16,17]. However, the used exposure times were often too long (30–60 s) to meet industrial demand. Moreover, these studies were focused on changes in viscoelastic and tribological properties after plasma treatment. Despite BOPP being one of the most abundant polymers utilized in the packaging industry, plasma activation by DCSBD for a shorter treatment time (1–5 s) has only been reported once [18]. However, the achieved results were discussed very briefly, and the operation parameters of the DCSBD plasma source have been upgraded since the publishing of these data. There is lack of systematic study of the DCSBD plasma effect on the surface properties of BOPP substrate.

Here, we investigate and compare the efficiency of routinely used VDBD plasma represented by industrial corona systems and its possible alternative, namely DCSBD, for standard industrial roll-to-roll BOPP foil processing. The DCSBD in a concavely curved configuration of the electrode system is used for surface activation of BOPP foil, and changes in surface characteristics are compared with those achieved after treatment by VDBD. Morphological changes, surface wettability, adhesion, chemical composition, and the stability of the achieved properties are investigated.

## 2. Materials and Methods

### 2.1. Material

Biaxially oriented polypropylene (BOPP) cigarette foil used for the experiments was provided by Chemosvit folie s.r.o., Svit, Slovakia. BOPP foil with a thickness of 25 μm and a square weight of 22.8 g m^−2^ was delivered in the form of a roll. BOPP foil of a width of 25 cm was treated by plasma and cut to the required size for a particular analysis.

### 2.2. Plasma Treatment

**Figure 1:**
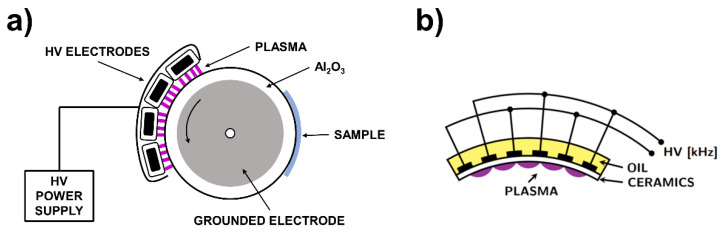
Schematic representation of used plasma sources: (**a**) VDBD electrode system and (**b**) concavely curved DCSBD electrode system.

The plasma treatment was carried out by two different dielectric barrier discharges generating nonequilibrium “cold” atmospheric plasma. Diffuse coplanar surface barrier discharge (DCSBD) in concavely curved configuration of the electrode system operating at atmospheric pressure in ambient air produces particularly diffuse “cold” plasma, while volume dielectric barrier discharge (VDBD) generates “cold” plasma solely in filamentary mode. A schematic description of both electrode systems is depicted in Figure 1. Detailed technical specifications of both plasma sources were discussed elsewhere [7]. Regarding DCSBD, the sample was attached to the roller at a distance of 0.3 mm from the electrode system. Input power was adjusted at a value of 400 W (the frequency of ~15 kHz), and the treatment speed was set at a constant value of 4.8 m min^−1^ corresponding to a treatment time of 1 s. Samples were exposed to plasma for 1, 3, 5, and 10 s applying the corresponding rotation cycles. VDBD operated in the following conditions: the input power was set to the value of 380 W, corresponding to the same square power density of 2.5 W cm^−2^ as the plasma produced by DCSBD. The average speed of cylinder rotation was 18 m min^−1^, and the distance between the sample and high-voltage electrodes was set to the value of 1 mm. Treated samples were stored in Petri dishes under the following laboratory conditions: temperature = 23 °C and humidity = 40%.

### 2.3. Analytical Methods

Imaging of surface morphology was performed using a Scanning Electron Microscope Mira3 (Tescan, Brno, Czech Republic) with a maximum resolution of 1 nm and a maximum magnification of 1,000,000. The detector of secondary electrons and accelerating voltage of 15 kV was used. The surface morphology analysis was conducted with magnification up to 50,000. To prevent charging of the sample, the BOPP foil surface was coated with 20 nm of the Au/Pd layer by sputter coater Quorum Q150R-ES (Quorum Technologies, Lewes, United Kingdom). The changes in surface roughness were measured using Atomic Force Microscope NTEGRA Prima (NT-MDT, Moscow, Russia) in a semi-contact mode. The Root Mean Square (RMS) roughness was estimated from the area 5 × 5 mm^2^ with resolution 512 × 512 px^2^ and scanning frequency of 1 Hz.

The contact angles (CA) of water, diiodomethane, and ethylene glycol were measured by a See (Surface Energy Evaluation) System analyzer (Advex Instruments, Brno, Czech Republic) using sessile droplets with volume 1 µL. The resulting values of CA were calculated as an average value from at least ten droplets taken at the BOPP surface exposed to plasma at different treatment conditions. Surface free energy (SFE) values were calculated using the Owens-Wendt regression model [19].

XPS analysis was performed by the spectrometer Axis Supra (Kratos Analytical Ltd., Manchester, United Kingdom) using monochromated AlKα radiation of energy of 1486.6 eV. Emitted photoelectrons were collected by an analyzer from a sample area of size 300 × 700 µm^2^ perpendicular to the sample surface. Because the samples are insulators, it was necessary to use a charge neutralizer electron source to compensate for sample charging during analysis. All spectra obtained under such conditions are shifted from the base position by a few eV to the lower binding energies. Therefore, it was necessary to perform energy calibration by shifting spectra according to a reference peak. Survey spectra were collected using an analyzer pass energy of 80 eV and high-resolution spectra for pass energy of 20 eV. The step size of the high-resolution spectra was 0.1 eV. Spectra calibration, processing, and fitting routines were completed using CASA software (trial version CasaXPS 2.3.16, CASA international nv, Olen, Belgium).

Static material testing machine Texture Analyser TA.XT *plusC* (Stable Micro Systems, Surrey, United Kingdom) was used for peel force measurements [20,21]. The 90° tape peel test for evaluating adhesion on plasma-treated BOPP foils was carried out using a peel fixture called “Rotating German Wheel” for continuous peeling off of the adhesive tape from the sample. Measurement was performed according to the FINAT test method no. 2. (a 90° peel adhesion test). The loading speed was set to the value of 10 mm min^−1^, and the load cell with a 50 N range was used for adhesion measurements. The sample was prepared by sticking a 19 mm wide -Scotch™ Magic™ adhesive tape (3M, St. Paul, MN, USA) on the BOPP foil sample and ensuring 10 passes over a taped area with a rolling pin. The evaluation of measured peel force was in a range from 20 mm to 70 mm, whereas measurement values for the initial length of 20 mm were discarded. The average peel force was calculated from 3 to 5 tests of samples treated in the same conditions [7].

## 3. Results

### 3.1. Surface Morphology

**Figure 2:**
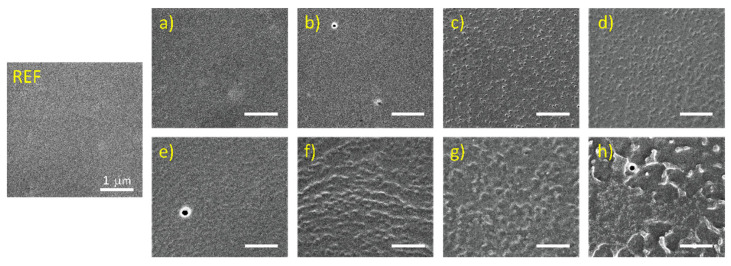
SEM images of BOPP foil treated by plasma at the different experimental conditions: (**a**–**d**) DCSBD at 1 s, 3 s, 5 s, and 10 s and (**e**–**h**) VDBD at 1 s, 3 s, 5 s, and 10 s.

Physicochemical interactions at the plasma–polymer interface can induce the etching of the polymer surface, which primarily affects the morphology of the surface. Therefore, morphological changes were monitored by scanning electron microscopy (SEM) and atomic force microscopy (AFM). The SEM image of the untreated BOPP foil surface (REF) depicted in Figure 2 demonstrates its very smooth and homogeneous nature at the micrometer scale. Lower plasma exposure times (1 and 3 s) in the case of DCSBD did not affect the surface morphology. After 5 s as well as 10 s of plasma treatment, we observed the formation of droplet-like structures with a diameter around 50–100 nm. Compared to the moderate effect of DCSBD on surface roughening, VDBD had a much more pronounced effect on the surface morphology. One second of plasma treatment showed mild structuring (Figure 2e) of surface, but a longer treatment time induced formation of droplet-like structures. However, the formed droplets had a size of around 200 nm (3 and 5 s in Figure 2f,g) or even larger (10 s in Figure 2h). Our results from SEM were quite different from other studies. Shekargoftar et al. [11] treated the PP/Al/PET-based laminated foil by DCSBD as well as the VDBD plasma source. The authors achieved the droplet-like structure at the PP side of the foil, after 3 s of plasma treatment by both plasma sources. Additionally, the droplets were enlarged due to merging after DCSBD treatment (up to 5 µm in diameter) in comparison to VDBD. A recent study by Janík et al. [16] demonstrated the formation of very similar structures after the treatment of PP specimens by coplanar DCSBD. However, in both mentioned studies, the size of droplets formed after treatment by DCSBD plasma was around 1–5 µm, which is much higher than our droplets possessing size in nanometers. In the case of PP/Al/PET-based laminated foil [11], these differences could be caused by the presence of a conductive Al layer, causing the parasitic microfilaments to burn perpendicular to the foil during DCSBD treatment, and also by the different nature of the used PP substrate, which relates to the manufacturing process as well as to the ratio of amorphous and crystalline regions on the surface. The degree of crystallinity and arrangement of crystalline and amorphous segments in polymer determine the resulting structuring of the surface after plasma treatment [22]. Plasma etching leads to the faster degradation of the amorphous phase in the BOPP structure, while crystalline regions become revealed, which defines the resulting roughening.

**Figure 3:**
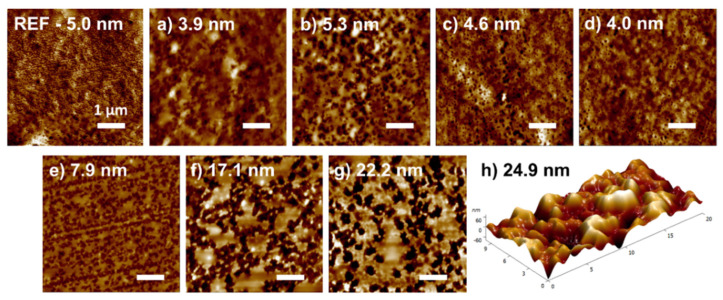
AFM images of BOPP foil treated by plasma at the different experimental conditions: (**a**–**d**) DCSBD at 1 s, 3 s, 5 s, and 10 s and (**e**–**h**) VDBD at 1 s, 3 s, 5 s, and 10 s. Values of roughness are inserted in the appropriate pictures.

The AFM images are depicted in Figure 3 with inserted values of RMS roughness. The results from the AFM measurement revealed the low degree of roughness of raw BOPP foil with a value of 5.0 nm comparable to other papers. Strobel et al. [23] observed RMS roughness values in the range 2.3–4.3 nm for various types of BOPP differing in orientation. Another paper by Chen et al. [24] measured RMS roughness of 3.8 nm, and Darvish et al. [25] published roughness of BOPP film with a value of 6.8 nm. Exposure to DCSBD only slightly influenced the RMS values, but the AFM images show negligible surface topography changes. Plasma treatment during the first 3 s (Figure 3a,b) induced the formation of hole-like structures even though the roughness seemingly did not change in comparison to reference. However, the formed holes possessed very low depth, which supported small differences in roughness. Longer treatment times (Figure 3c,d) resulted in a decrease in roughness, but values varied in the range of 4–4.6 nm, which represented a negligible change. In accordance with the SEM images, plasma treatment by VDBD induced more pronounced changes in topography and roughness. With increasing plasma exposure times, roughness gradually increased (Figure 3e–h) to the highest value of 24.9 nm after 10 s of VDBD plasma treatment. Wang et al. [26] used atmospheric-pressure dielectric barrier discharge to generate the air plasma for PP treatment and achieved roughness values from 15.3 nm to 55.3 nm depending on different treatment conditions. Oravcová et al. [18] monitored the effect of atmospheric-pressure plasma generated by DCSBD on surface characteristics of monoaxially oriented PP. They achieved a roughness increase to value 24 nm after 5 s of plasma treatment. However, the BOPP in our case cannot be directly compared with other PP substrates, because the manufacturing by stretching in two directions provides different properties than other types of PP. BOPP was treated by DBD in the study of Chen et al. [24], where they observed a change in roughness from the initial 3.8 nm to 7.2 nm after 3 s of treatment which corresponds to our value after 1 s of VDBD treatment.

Moreover, morphological changes monitored on the BOPP surface treated by VDBD induced changes in the optical properties of BOPP foil. The fully transparent foil lost its transparency after 3 s of exposure, and we monitored the formed haze, which intensified with the plasma treatment time. Haze is caused by light scattering, which can originate from the bulk of the material as well as from its surface. Since the DCBSD treated samples did not change their optical properties, the haze visible on VDBD treated samples is probably connected to the creation of hole-like structures (Figure 3f–h), which can cause light scattering. The most probable explanation for such structures is the penetration of filamentary plasma through the upper layer of the surface. Moreover, the generated droplet-like structures with sizes between 0.2 and 1 µm corroborated these findings. Surface structures possessing dimensions similar to the visible light wavelength induce Mie scattering resulting in the milky appearance of the plasma treated foil [27].

### 3.2. Wettability and Ageing Study

**Figure 4:**
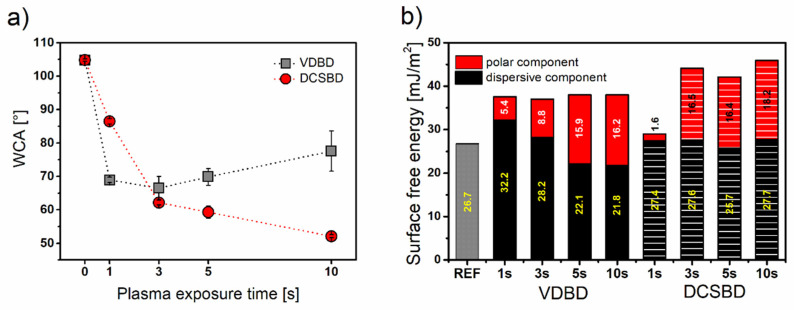
Graphs of WCA and SFE measurements: (**a**) WCA values measured immediately after plasma exposure of BOPP foil by VDBD (grey square) and DCSBD (red circle) for different plasma treatment times (1, 3, 5, and 10 s); (**b**) comparison of surface free energy (SFE) of BOPP foil treated by VDBD and DCSBD with inserted values of polar and dispersive component of SFE.

Regarding the hydrophobic nature of polypropylene, the wettability of pristine BOPP film is very low, which agrees with the water contact angle (WCA) value of 104.8 ± 0.4° and surface free energy (SFE) value of 26.8 mJ m^−2^. The development of WCA after plasma treatment is depicted in Figure 4a, and changes in SFE are shown in Figure 4b. The treatment of BOPP surface by both plasma sources, coplanar and volume DBD, improved the wettability already after 1 s of plasma exposure. In the case of DCSBD, increased plasma exposure time resulted in gradually decreased WCA until achieving the lowest value (52.1 ± 0.5°) after 10 s of treatment. In comparison, VDBD resulted in decreasing WCA to 68.9 ± 0.8° already after 1 s. The lowest WCA was observed after 3 s of treatment (66.5 ± 3.5°), followed by an increase in WCA with prolonged plasma exposure time. The rising of WCA after high plasma exposure times (10 s) can be explained by roughening of the surface after the VDBD treatment caused by etching. Although, etching usually occurs at higher exposure times (order of minutes), in the case of destructive VDBD filamentary plasma, it is possible that 10 s is sufficient for surface roughening and undesirable hydrophobization. The study on the plasma treatment of selected polyolefins revealed how an appropriate combination of microscale features on the surface with plasma-etched nanoscale roughness can regulate the wettability of the substrate [28].

The SFE of pristine BOPP mainly comprised a dispersive component representing 26.7 mJ m^−2^ from 26.8 mJ m^−2^ of total SFE value. The absence of a polar component corresponds to the fully hydrocarbon structure of BOPP. Oxidation of the BOPP surface induced by plasma treatment resulted in an increase in SFE for both plasma sources (Figure 4b). Treatment by VDBD in all exposure times led to similar SFE values of 37–38 mJ m^−2^. Additionally, the polar component gradually rose with the increased plasma exposure time from 5.4 mJ m^−2^ to 16.2 mJ m^−2^ proving the high effect of atmospheric cold plasma on the polar part of SFE. The increasing polar component represents the formation of polar functional groups on treated BOPP due to the presence of oxygen and nitrogen in air. The air humidity and hydrogen abstraction from the polymer chain allows the formation of hydroxyl radicals in the gas phase and causes the formation of free radicals [29]. The free radicals provide further reaction of the activated surface with reactive oxygen and nitrogen species present in air plasma. The change in dispersive component is related mostly to the presence of nonpolar functional groups. Furthermore, the changes in surface morphology also contribute to the dispersive component. The increase in the dispersive component of SFE after the short VDBD plasma treatment was observed on PP in Shekargoftar et al. [11], followed by a decrease to the reference value after 5 s.

In comparison, DCSBD was more efficient in surface activation showing higher values of SFE (42–45.9 mJ m^−2^) except for the exposure time of 1 s where only 29 mJ m^−2^ was achieved. Moreover, atmospheric plasma generated by DCSBD had a more pronounced impact on the polar part of SFE, which reached values in the range of 16.4–18.2 mJ m^−2^. The polar component of surface energy represents the highest contribution to the total value of SFE. The increase in and further stabilization of the polar component of SFE were achieved after 3 s of DCSBD treatment. The dispersive component altered negligibly after the DCSBD treatment reflecting the small changes in surface morphology.

**Figure 5:**
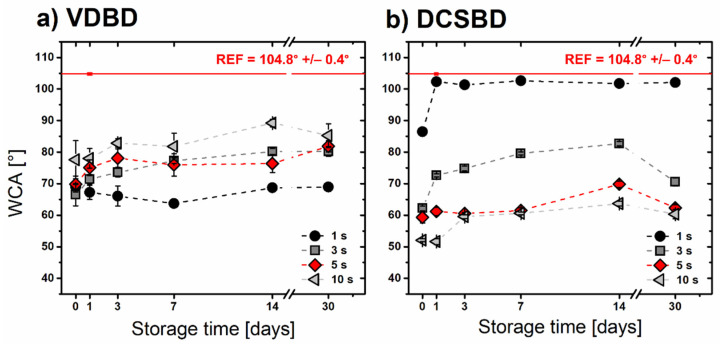
WCA development during the storage of BOPP foils under laboratory conditions: WCA values dependent on storage time after the plasma treatment by (**a**) VDBD and (**b**) DCSBD.

Enhancement of surface wettability after the plasma treatment is not fully permanent. The rate of hydrophobic recovery of the activated surface depends on many factors, such as the chemical nature of the substrate, storage conditions, as well as the used plasma source. Monitoring the WCA changes over time represents a great tool for investigating the stability of plasma-induced changes. WCA development during the 30 days of storage under laboratory conditions is illustrated in Figure 5. Surprisingly, the ageing effect for the samples treated by VDBD was very slow. WCAs measured on BOPP sample exposed to VDBD plasma for 1 s maintained the stable contact angle during the whole monitoring time. Similar behavior was observed by Borcia et al. [30] for HDPE treated by filamentary type of DBD. Compared to other hydrocarbon polymers (polystyrene and polymethylpentene), the HDPE surface stayed stable for two weeks. On the contrary, the BOPP sample exposed to DCSBD for 1 s recovered to the reference WCA value within the first 24 h. Further, VDBD samples treated for 3 s and longer experienced slight hydrophobic recovery during the first 3 days of storage. After a month of storage, all VDBD samples remained hydrophilic. Similar effects were observed in the case of 3–10 s DCSBD treated samples. However, after the month of storage, the WCA values were lower for DCSBD than for VDBD treated samples.

### 3.3. Peel Force

**Figure 6:**
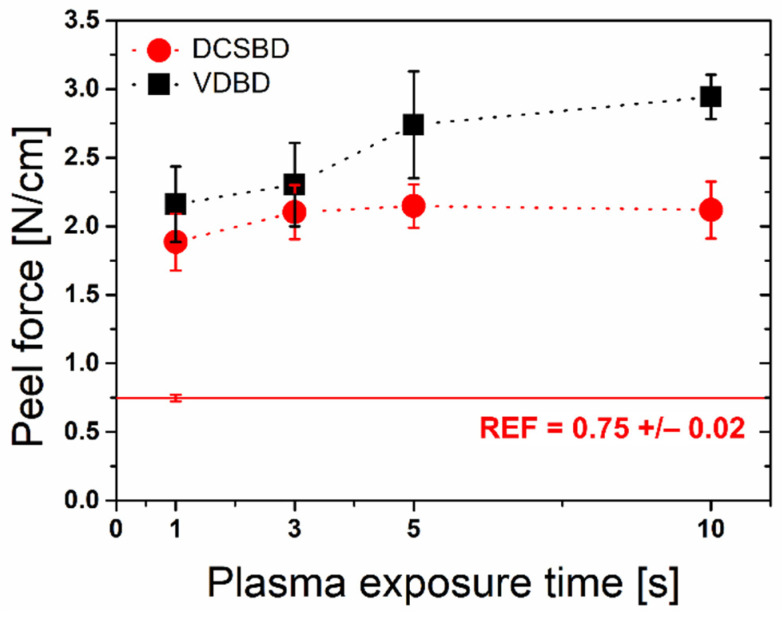
Development of the peel force of the BOPP surface after plasma treatment.

The peel force improvement of the BOPP surface was observed after the treatment by both plasma sources (Figure 6). The reference value of 0.75 N cm^−1^ almost tripled after 1 s of VDBD and rose with increased treatment time. The high error values of peel force are often related to the nonuniform treatment of large-area surfaces by VDBD [31]. In our case, these variations could be the results of surface topography and roughness changes, which also contributed to higher peel force values for VDBD compared to less invasive diffuse plasma generated by DCSBD. However, the adhesion improvement of the BOPP surface corresponds with the polar component increase after VDBD plasma treatment, which was also observed for DCSBD treated samples. After 3 s of treatment by DCSBD, the peel force value stabilized, and it did not change with prolonged treatment. In contrast, Bhat et al. [32] observed a decrease in peel force during the first 60 s of RF plasma exposure. The expected increase occurred after plasma exposure time on the order of minutes. The postponed effect of plasma treatment was explained as plasma cleaning of commercially manufactured and contaminated BOPP surface prior to surface modification. As a result, the time needed for BOPP adhesion improvement in the case of RF plasma was significantly longer than in our case. These results indicate the relation of BOPP adhesive properties with the formation of polar functional groups and surface roughness [5,32,33].

### 3.4. Surface Chemical Analysis

Improved wettability after plasma treatment indicates increased hydrophilicity of the BOPP surface related to the formation of polar functional groups. Chemical changes on the plasma-treated samples were monitored by X-ray photoelectron spectroscopy (XPS). The atomic composition of untreated and plasma-treated BOPP foil observed by XPS is summarized in Table 1. The untreated BOPP foil contained 95% carbon and 5% oxygen, which is in good accordance with other studies using various PP substrates [15,26,34,35]. The presence of oxygen on the raw BOPP surface suggests organic contamination or low-level surface oxidation. The treatment by both plasma sources induced an increase in oxygen concentration to 20% after 1 s. Further increasing plasma exposure time led to the higher oxygen content with the highest level at 28% for the sample treated by VDBD at 10 s. Otherwise, achieved oxygen contents were comparable for both plasma sources, reflecting the similar level of surface oxidation for diffuse and filamentary plasma. Compared to the study of Saranko et al. [15], where they observed 23.6% of oxygen content after 60 s of plasma treatment by DCSBD (pristine ~5.3%), we proved that a few seconds of plasma treatment were sufficient for surface activation of hydrophobic polymers. Despite the use of ambient air as a working gas for experiments, nitrogen atoms appeared at the BOPP surface in a negligible concentration (1–2.2%). A similar outcome was also monitored in other studies [29,36,37]. Dorai and Kushner explained the poor incorporation of *N*-based functional groups due to the low reactivity of N atoms towards the plasma-treated PP surface. They described in detail the mechanism of PP surface functionalization under the industrial corona treatment in humid air resulting in the formation of alcohol, carbonyl, carboxy, and peroxy groups.

The deconvolution of C1s high-resolution spectrum of the reference sample consisted dominantly of C–C/C–H bonds typical for a BOPP structure. Further, the residual quantity of the C–O bonds originating from the manufacturing process was revealed. After the air plasma treatment, the concentration of C–O bonds increased from an initial 6% and saturated at a value of approx. 15% for all used plasma treatment conditions. This can be explained by quenching of alkoxy radicals formed at the PP backbone after H-abstraction, which results in the formation of C–O groups. Other alkoxy radicals undergo the β-scission to yield C=O groups. In our study, the C=O and O–C=O bonds were formed right after the short plasma treatment by both plasma sources, and their concentrations increased with time. The level of C=O bonds was slightly higher for samples treated by VDBD as well as the amount of O–C=O groups. The O–C=O groups achieved higher values for all plasma exposure times in the case of VDBD treatment. The air humidity also plays a great role in the concentration of formed functional groups [29]. However, investigating the influence of relative humidity on chemical changes after plasma treatment of BOPP surface was outside of the scope of this study. Borcia et al. [30] observed comparable chemical changes on the surface of hydrocarbon polymers after treatment by air VDBD plasma having similar operating conditions as our experiment. Oxidation of poly(ethylene) (PE) was more efficient than in the case of branched poly(methylpentene) (PMP) in terms of functional groups concentration. In our case, after the corresponding treatment time, we observed 14–15.3% of alcohol groups presenting the lower level compared to 18–19.2% for PE and PMP in the mentioned study. However, the achieved concentrations of carbonyl and carboxyl functionalities in our experiment were between PE and PMP. Considering the structure of monomer units in particular polymers (PE, PMP and PP), oxidation of PE consisting fully of –CH_2_– bonds was faster than in the case of branched PMP and PP. According to the proposed mechanism for hydrocarbon oxidation in plasma [29], initial H-abstraction from the surface depends on its position in the polymer backbone. Although the probability of abstractions follows the order: H_tert_ > H_sec_ > H_pri,_ the most reactive tertiary H present in PP and PMP is hindered by less reactive H from –CH_3_ groups. This could be the reason for the more rapid oxidation of PE consisting solely of secondary H.

The polar functional groups contribute to the polar component of SFE and increased wettability of the BOPP surface. However, the respective XPS data do not reflect the corresponding WCA results. The similar behavior monitored by Borcia et al. [30] explains the discrepancies between WCA and XPS results based on the different effective depths analyzed by these two techniques.

**Figure 7:**
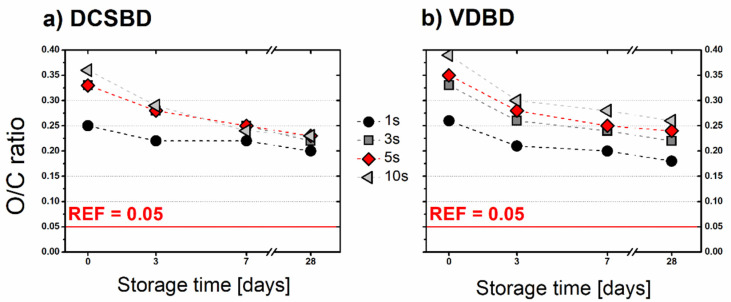
Development of the O/C ratio during the storage of BOPP foils under laboratory conditions: O/C ratio plotted against the storage time for (**a**) DCSBD and (**b**) VDBD for all plasma treatment conditions.

Additionally, XPS analysis was employed to monitor the stability of surface oxidation during the storage of samples under laboratory conditions. In Figure 7, the O/C ratio is plotted against the storage time. Ageing curves of BOPP samples treated by DCSBD for 3–10 s follow a similar decreasing trend and end up around value 0.22. In general, the O/C ratio values achieved right after the treatment decreased by 31–39% after 28 days of storage. In comparison to these samples, the sample treated for 1 s possessing the lowest oxidation exhibited a less steep trend of ageing (20%). The O/C ratio of VDBD treated samples after 28 days of storage were very similar to those achieved in the case of DCSBD (the decrease by 30–34%). Leroux et al. [36] investigated the development of the O/C ratio on the PP surface treated by DBD plasma during the 30 days of ageing. Surprisingly the ageing process was slower than in our case (17–23%); however, they observed much lower values of O/C ratios (0.12–0.16). A similar trend of O/C ratio decrease during ageing was also observed for oxygen containing functional groups proportional to C-C bonds.

## 4. Conclusions

In the present work, we investigated the impact of VDBD and DCSBD plasma treatment on the surface characteristics of BOPP foil. Considering the surface morphology, the filamentary plasma produced by VDBD had a more destructive effect than the diffuse plasma generated by DCSBD. The smooth nature of BOPP foil remained unchanged during the first 3 s of DCSBD plasma treatment, whereas VDBD caused surface roughening after 1 s of treatment. Moreover, plasma exposure time higher than 1 s in the case of VDBD induced undesirable haze. Although DCSBD treatment at 5–10 s resulted in the formation of droplet-like structures, the foil remained transparent. Improvement in wettability was achieved for both plasma sources. However, exposure to VDBD plasma longer than 3 s led to a WCA increase perhaps due to surface roughening. Increased roughness after VDBD treatment also contributed to enhanced adhesion, where longer treatment time (5–10 s) caused an increase in peel force. Nevertheless, adhesion improved after 1 s of treatment by both plasma sources, indicating the direct relation of BOPP adhesive properties with the formation of polar functional groups. Surface oxidation was achieved after 1 s of plasma exposure and increased with the plasma treatment time. Observed oxygen contents were comparable for both plasma sources reflecting the similar level of surface oxidation for diffuse and filamentary plasma. Considering the WCA values as well as the O/C ratio development during the month of storage, the acquired surface properties slightly recovered without achieving initial characteristics. BOPP foil remained hydrophilic after the treatment by both plasma sources; however, the WCA after a month in the case of DCSBD were lower than for VDBD treated samples. These data show that BOPP foil requires treatment by diffuse plasma of DCSBD longer than 1 s for sufficient surface oxidation. BOPP treated at 3 s by both plasma sources achieved similar surface activation. However, considering the surface morphology, VDBD treatment longer than 3 s induced structural changes in the microscale which led to optical haze, whereas DCSBD treatment retained the transparency of the foil. Although these results prove a similar efficiency of diffuse and filamentary plasma on BOPP surface activation, it supports the high potential of DCSBD technology to be part of industrial systems as it is gentler to sensitive polymeric surfaces.

## Figures and Tables

**Table 1:** The atomic concentration and relative area of C1s chemical bonds of the BOPP surface analyzed by XPS measurement after treatment by DCSBD and VDBD plasma sources.

		Atomic Concentration [%] ^1^			O/C Ratio	Functional Groups Concentration [%] ^2^			
		C	O	N		C–C/C-H	C–O	C=O	O–C=O
						284.8 eV	285.9 eV	287.5 eV	289.3 eV
	REF	95	5	-	0.05	94.2	5.8	-	-
DCSBD	1 s	80	20	<1	0.25	76.5	15.1	6.7	1.7
	3 s	74	24	1.8	0.33	71.5	14.3	8.1	6.1
	5 s	73	24	2.2	0.33	69.2	14.2	9.1	7.5
	10 s	72	26	1.7	0.36	65.8	15.9	9.8	8.6
VDBD	1 s	79	20	<1	0.26	73.7	14.0	7.1	5.3
	3 s	74	25	1.0	0.33	66.5	15.3	9.3	8.9
	5 s	73	26	1.1	0.35	65.7	15.2	9.1	10.0
	10 s	71	28	1.2	0.39	62.3	15.3	9.6	12.9

^1^ Estimated from survey spectra. ^2^ Estimated by deconvolution of C1s high-resolution spectra.

## Data Availability

Not applicable.
